# *Burkholderia cepacia* Complex Vaccines: Where Do We Go from here?

**DOI:** 10.3390/vaccines4020010

**Published:** 2016-04-15

**Authors:** Gonzalo A. Pradenas, Brittany N. Ross, Alfredo G. Torres

**Affiliations:** 1 Department of Microbiology and Immunology, University of Texas Medical Branch, Galveston, TX 77555, USA; gopraden@utmb.edu (G.A.P.); brross@utmb.edu (B.N.R.); 2 Sealy Center for Vaccine Development, University of Texas Medical Branch, Galveston, TX 77555, USA

**Keywords:** *Burkholderia cepacia* complex, vaccine, cystic fibrosis

## Abstract

*Burkholderia* comprises a wide variety of environmental Gram-negative bacteria. *Burkholderia cepacia* complex (Bcc) includes several *Burkholderia* species that pose a health hazard as they are able to cause respiratory infections in patients with chronic granulomatous disease and cystic fibrosis. Due to the intrinsic resistance to a wide array of antibiotics and naturally occurring immune evasion strategies, treatment of Bcc infections often proves to be unsuccessful. To date, limited work related to vaccine development has been performed for Bcc pathogens. In this review, we have gathered key aspects of Bcc research that have been reported in recent years related to vaccine efforts, virulence, immune responses, and animal models, and use this information to inform the research community of areas of opportunity toward development of a viable Bcc vaccine.

## 1. Introduction

*Burkholderia* comprises a wide variety of environmental, Gram-negative, obligate aerobes, commonly found in soil and ground water [1]. *Burkholderia cepacia* complex (Bcc) is a subgroup within the *Burkholderia* genus, which currently [2] comprises 20 species known to be opportunistic pathogens and causative agents of respiratory infections in patients with cystic fibrosis (CF) and chronic granulomatous disease (CGD) [3,4,5]. The incidence of Bcc bacteria in CF patients is close to 2.6% in the USA and 3.8% in the UK [6,7], with *B. multivorans* and *B. cenocepacia* causing the majority of infections [8]. Although *Pseudomonas aeruginosa* is the most common CF-associated pathogen, Bcc infections pose a higher risk because CF patients experience a rapid decline in pulmonary function [9]. In some cases, CF-related Bcc infection results in the “cepacia syndrome”, which consists of acute [1] pulmonary deterioration with bacteremia and necrotizing pneumonia, that is often lethal to CF patients [10].

Additionally, Bcc cause infections outside CF and CGD populations. In fact, worldwide outbreaks of nosocomial infections were reported in the past few years [11,12,13]. These infections are usually due to the contamination of medical supplies [14], such as nasal sprays and ultrasound gels, and affect a wide range of risk groups [11]. These health risks are particularly problematic, since Bcc has several features that make it difficult to treat. These characteristics range from high and wide antibiotic resistance, biofilm production, resistance to antimicrobial peptides, and the ability to adhere, enter, and survive intracellularly in human respiratory epithelial cells, neutrophils, and macrophages [10,15,16].

## 2. Bcc Virulence Factors

Since Bcc treatment presents a complex scenario, research has been focused on understanding the mechanisms of pathogenesis aimed at providing new treatment avenues [10,17]. Studying virulence factors may be useful for vaccine development, since it often identifies different protein targets that could potentially be immunogenic or may serve as promising candidates for inactivation in the development of live attenuated vaccines.

In this context, quorum sensing may be an interesting target for the generation of attenuated vaccines. This is because it mediates the expression of multiple genes in bacteria, and appears to be central in the regulation of virulence-related genes in Bcc. Specifically, the *cepIR* and *cciIR* quorum-sensing systems have been implicated in pathogenesis, as demonstrated in a rat chronic lung infection model [18]. These systems have also been shown to be required for bacterial motility [19], biofilm stability [20], production of virulence factors [21], and protease expression [22].

Bacterial biofilm production is closely associated with quorum sensing. Biofilms are complex microbial communities that are able to adhere to different surfaces and enhance bacterial virulence and antimicrobial resistance [23]. *In vitro* biofilm formation is well documented for *B. cenocepacia* and has been shown to be affected by several systems, such as quorum sensing [20], alternative sigma factors [24], exopolysaccharide synthesis [25], motility, and iron availability [26]. In the case of CF patients, Bcc was initially thought to reside in the lungs where it forms biofilms that communicate with other CF pathogens via quorum sensing [27]. However, subsequent findings disputed this notion after detecting *B. cenocepacia* and *B. multivorans* in CF lungs recovered from transplant recipients. The study found that both strains were either within phagocytes or mucus, but not in biofilms [28]. Since this area in the Bcc literature is controversial, vaccine development strategies that target biofilm formation may not be ideal at this time.

Specialized secretion systems are among the more interesting virulence factors, and can also be used for vaccine development. During bacterial infection, the secretion of effector molecules into the host cell can elicit a wide range of effects that contribute to disease, including disrupting or modifying host cell processes and responses; hence, its importance in bacterial pathogenesis [29]. In the context of Bcc, several types of secretion systems (TSS 2, 3, 4 and 6) have been reported to play different roles in *B. cenocepacia* virulence.

Specifically, type 2 secretion system (T2SS) proteins ZmpA and ZmpB are necessary for *B. cenocepacia* virulence and intra-macrophage survival [30]. Similarly, the type 3 secretion system (T3SS) has proven essential for bacterial survival in the murine model of chronic infection [31]. The type 4 secretion system (T4SS) of *B. cenocepacia* is required for bacterial escape from endosomes and aids in its intracellular survival in epithelial cells and macrophages [32]. Finally, evidence suggests that the type 6 secretion system (T6SS) present in *B. cenocepacia* might have a role in pathogenesis, since three mutants belonging to the T6SS gene cluster were attenuated in the murine chronic infection model [33]. Since the structural and secreted effectors of many other pathogens have been identified as immunogenic, the presence of several secretion systems in Bcc provides many possible immunogenic targets for a subunit vaccine [34]. Their presence also allows for many mutation candidates for the generation of a live attenuated vaccines [34,35].

## 3. Bcc Animal Models

In the search for virulence determinants for Bcc species, many virulence models have been developed. An increasingly popular model uses invertebrates because they are simpler and faster options compared to traditional mammalian models. Invertebrate models are not useful for vaccine testing; however, they often produce results that correlate well with those from murine models of infection. To date, these models have been used extensively to test for pathogenesis [36,37] and to evaluate the efficacy of novel antimicrobials for a variety of microorganisms [37,38,39]. For instance, the fruit fly, *Drosophila melanogaster*, was successfully used to determine the virulence of *B. cenocepacia* K56-2 [40] and also used for screening of Bcc-attenuated mutants to identify novel virulence factors. Similar considerations apply to the nematode *C. elegans* [41].

This is also the case for the wax moth, *Galleria mellonella*, a larvae infection model that is useful for virulence determination and the evaluation of novel antibiotic/antibacterial therapies, such as the effect of KS4-M phage therapy [42]. KS12 phage/meropenem therapy has been also successfully tested against *B. cenocepacia* in *G. mellonella,* and results point to phage therapy as a possibly effective treatment approach [43].

A more clinically relevant approach for the development of Bcc vaccines is the use of murine models. For example, BALB/c mice have been used for intraperitoneal and nasal infections, allowing evaluation of bacterial clearance, persistence, and overall clinical differences between Bcc genomovar strains [44]. Although the intraperitoneal infection lacks clinical relevance for respiratory Bcc infections, it is a useful model in the examination of Bcc persistence in the spleen. In contrast, intranasal, intratracheal, and aerosol models are ideal for testing mucosal immunity and represent respiratory infections.

Since Bcc infections are linked mainly to CF patients, gene knock-out (KO) mice have been developed to study these infections in the context of a CGD- or CF-like model. The development of gp91phox−/− strain of mice, which mimic chronic granulomatous disease, has allowed for the study of Bcc survival in infected neutrophils, and the results have been used to assess short-term virulence determinants [45]. Another useful model to study infection is the *Cftr*−/− mouse, which replicates the gene loss found in CF patients. These mice are used for studies of virulence [46], Bcc persistence [47], cell adherence [48], and to examine the inflammatory response [46]. This model has also been used to test the efficacy of gene complementation therapy on CF lung infections [49]. When combined with chronic infection models, in which Bcc bacteria are embedded into agar beads and then introduced intratracheally, a lower respiratory infection model is obtained and was used to examine co-infections with *P. aeruginosa* and the effects on lung pathology [46].

It is generally well accepted that CF-deficient mice do not reproduce the chronic bacterial infection typically seen in CF patients [45]. To address this issue, alternative *Cftr*-defective animal models, such as ferrets and pigs, have been developed. Ferrets have been used extensively to study viral respiratory infection, mainly due to the physiological similarities between the human and ferret lung [50]. *Cftr*−/− ferrets display many of the characteristics of human CF disease, including airway chloride transport defects and submucosal gland fluid secretion [51], as well as evidence of spontaneous lung infections early in their development [51]. These ferrets have also been successfully used as a model for vaccine development against several respiratory viruses [52]; however, its usefulness with bacterial vaccines remains to be demonstrated.

Similarly, the *Cftr*−/− pig model also looks promising. CF pigs develop lung disease within the first few months of life, characterized by airway inflammation, mucus accumulation, and spontaneous bacterial infection [53]. When facing bacterial challenge, CF pigs fail to kill bacteria, suggesting impaired innate immunity in the lung, similar to that of CF patients [54]. Thus, this model could be useful in vaccine development since the immune system of the pig closely resembles that of humans. For instance, the pig possesses dendritic cell populations with TLR responses as well as Th17 cells [55]. Moreover, the wild-type pigs have been used to test vaccines against pathogenic respiratory bacteria [56], making it a very attractive option for Bcc vaccine development.

In summary, vaccine studies would likely be initiated in BALB/c mice before advancing to immune compromised models. Further elucidation of a vaccine might then be performed in gp91phox−/− and *Cftr*−/− small animal models, which may be useful to predict effectiveness in the presence of the CF or CGD gene mutations. Ideally, the most suitable models to test vaccine efficacy are *Cftr*−/− ferrets and, even more so, *Cftr*−/− pigs.

## 4. Immune Response

Evaluating the immune response elicited by the Bcc species has proven difficult for several reasons: (1) they reside in the mucin layer or within intracellular environments; (2) they possibly form biofilms; and (3) they can modulate and overcome the hosts’ immune responses. Further, CF patients have abnormal immune regulation, which adds another layer of complexity [72].

One of the most notorious aspects of Bcc infections, especially in CF, is that *Burkholderia* species activate Toll-like receptors (TLR). LPS from *B. cenocepacia* is able to elicit a nine-fold higher pro-inflammatory response compared to that of *P. aeruginosa* [57]. Another *B. cenocepacia*, strain K56-2, does not elicit a potent LPS response, but is still able to stimulate a strong, flagellin-associated, TLR5 response [58]. Further, some strains of *B. cenocepacia* can bind to TNF receptor 1 (TNFR1), leading to more robust IL-8 expression, compared to those that cannot bind to the receptor [59]. The activation of TNFR1, along with TLR stimulation, promotes a pro-inflammatory environment that may be responsible for excessive neutrophil recruitment and impaired clearance, which is believed to ultimately lead to tissue damage [58,59]. Thus, the bacteria’s ability to modulate the host immune system could be one of the major hurdles in controlling and clearing the infection; however, further studies are required to fill the current gaps in knowledge.

It has been proposed that some Bcc strains are able to enter and survive in different pulmonary cell types *in vitro* [60,61,62,63]. They have also been shown to be taken up by human and murine macrophages [32,64,65]. It has been suggested that Bcc isolates are usually contained within vacuoles inside the macrophage [32,64,65]. Bcc engulfment by macrophages also cause a delay in the fusion and maturation of lysosomes, which is required for the survival of *B. cenocepacia* within this cell type [64]. It has been postulated that the internalization pathway possibly enables the bacteria to activate genes that confer resistance to the survival within the macrophage.

In the case of epithelial cell survival, it has been reported that, in IB3 (CF phenotype) cells, *B. cenocepacia* is internalized through the endocytic pathway, where it resides transiently in early endosomes, and then escapes from late endosomes. The organism can then be found in autophagosomes, and at the end of this pathway it is found replicating in the ER [60]. Alternatively, *B. multivorans* appears to access epithelial cells through paracytosis and cell destruction. Entrance, survival, and replication were also observed for *B. cenocepacia* LMG 16656 and *B. multivorans* in 16HBE14o- and CFBE41o-epithelial cells [66]. These findings suggest that Bcc possess several strategies for interacting with host cells and potentially evade the host defenses during chronic infections. These aspects of Bcc survival are important when designing a vaccine to ensure the immune response that is generated is able to inhibit these pathways so as to keep the pathogen from reaching its niche.

Another important aspect to consider during vaccine design is the optimal balance of Th1 and Th2 responses required for effective pathogen clearance. The bias, or balance, between Th1 to Th2 activation, can be examined by obtaining the ratio of IgG1 and IgG2 antibody titers. When bias occurs, different immune pathways are utilized. For example, a Th1 bias elicits a cell-mediated response, while Th2 induces a humoral immune response [67]. When it comes to vaccination, the host’s genetic background, dose, route, and adjuvant use have important effects on the type of immune response generated [68]. In the case of Bcc vaccines, the type of response needed to clear the pathogen are still not fully understood, making it difficult to generate a protective vaccine. BALB/c mice intraperitoneally immunized with Bcc-conserved proteins, Linocin and OmpW, displayed either a robust Th1 (Linocin) response that significantly reduced *B. cenocepacia* and *B. multivorans* burden and dissemination to the spleen, or a mixed Th1/Th2 (OmpW) response associated with bacterial reduction in the lungs and adjuvanticity effect [69]. Nasally immunized mice conferred different degrees of protection against *B. cenocepacia* and *B. multivorans* [70,71], and also elicited differing Th1/Th2 bias. As observed in CD-1 mice immunized with *B. cenocepacia* OmpA alone results in a Th2 bias response that does not significantly reduce bacterial burden, but when adjuvented with nanoemulsion it produced a Th1/Th2 balanced response that resulted in moderate reduction in bacterial burden [70].

Other observations seem to show that a Th1 response may be critical to fight *B. cenocepacia* infections. For instance, in a BALB/c mice model, the lack of IFN-γ production allowed *B. cenocepacia* to persist in the spleen, implicating the importance of a Th1 bias response [44]. However, during respiratory infection with *B. cenocepacia* BC7 in mice, a pronounced Th1 response occurs, yet the bacteria are not cleared [72]. These data indicated that a specifically targeted Th1 may be useful.

An added layer of complexity occurs when treating CF patients because they have an immune phenotype that appears to be skewed towards Th2 responses (with reduced Th1 responses) [73,74]. However, in some models it has been observed that a Th1 response may be important for the clearance of CF-related pathogens, including *B. cenocepacia*. Therefore, eliciting a Th1 response via adjuvantation may be beneficial to help balance the Th1/Th2 response and produce a more efficient immune response in the Th2 CF lung.

The Th17 response, which is produced by a relatively “recently” described CD4^+^ T Helper linage [75], is another feature of the immune response worth exploring for Bcc. Th17-associated cytokines are believed to play a role in host defense of mucosal surfaces, particularly in the lungs, through the recruitment of neutrophils to protect the host against bacterial pathogens. A strong Th17 response has been associated with the successful use of a *Klebsiella pneumoniae* subunit vaccine [76] and in the protection elicited by a live attenuated *Yersinia pestis* vaccine [77]. Similarly, Th17 responses have been associated with vaccine protection against other lung pathogens, such as *P. aeruginosa* and *Mycobacterium tuberculosis* [78,79]. Based on these studies, exploring Th17-eliciting vaccines may represent an attractive opportunity to combat Bcc. However, safety concerns often arise for vaccines that target production of a strong Th17 response. First, the effects of a Th17 inducing vaccine have not been well-defined in humans and Th17 T cells have the ability to cause tissue damage via a Th17 exacerbated pro-inflammatory response. This topic is especially important for CF patients because CF lung damage and decline in function is tightly associated with an exacerbated inflammatory response during chronic infections [80]. In fact, a pathogenic role of Th17 cells has been proposed, since increased levels of IL-17 and IL-23 [81] are detected during lung exacerbations. As research progresses, these issues may be solved, given that “protective” and “pathogenic” Th17 cells seem to have different phenotypes. Advances have been made to differentiate between these two populations [82], thus providing new opportunities to understand how to modulate specific Th17 responses.

## 5. Vaccines

Throughout the history of Bcc vaccine development, the main focus has been largely on protein subunit vaccines. Recently, other options, such as those incorporating complex carbohydrates into vaccine formulations, are making their way onto the scene as glycoconjugate platforms. With advances in vaccinology, alternative vaccine formulations must be explored, but some approaches have been neglected to this point. For example, there is no literature on the evaluation of potential live attenuated or heat-killed whole cell-based vaccines. In this section, we address the progress, both positive and negative outcomes, as well as future proposed directions on Bcc vaccines.

### 5.1. Subunit Vaccines

Currently, FliC (the flagella major subunit) and LolC (ATP-binding cassette system protein) have been proposed as potential subunit vaccine candidates in non-Bcc species due to their ability to elicit long lasting immune memory in mice and because they possess multiple type-II HLA epitopes [83,84]. Even though these proteins are conserved in Bcc, neither of these vaccine candidates have been tested in a Bcc infection model. If FliC and LolC are able to provide an immune response similar to that demonstrated with *B. mallei* and *B. pseudomallei* challenge, they may provide significant protection by reducing the bacterial burden promoting clearance of the pathogen [85]. In the case of *P. aeruginosa*, its elastase peptide has also been shown to participate in lung injury. Vaccination against elastase peptide fragments conjugated to keyhole limpet hemocyanin resulted in reduced lymphocyte infiltration and no apparent histopathological changes after *B. cepacia* Pc715j infection [86], suggesting some degree of cross-protection.

Linocin and OmpW, from *B. multivorans* [69], represent additional potential vaccine candidates [69]. Vaccination in mice with both antigens elicited noticeable serological responses, induced mixed Th1/Th2/Th17 responses, and also conferred some degree of protection against both *B. cenocepacia* and *B. multivorans* challenge. Interestingly, this protection was greatest against *B. cenocepacia* over *B. multivorans*, resulting in reduced lung bacterial load in mice immunized with OmpW and, to a greater extent, with Linocin.

More successful vaccination has been observed with un-purified outer membrane proteins (OMP) from *B. multivorans* and supplemented with the mucosal adjuvant adamantylamide dipeptide. These mixture resulted in a 12-fold increase in IgG, a 6-fold increase in mucosal IgA, and reduced bacterial burden and lung pathology. The OMPs vaccine provided immunity and a reduced burden against *B. multivorans* and moderate cross-protection to *B. cenocepacia* [71]. Recently, work building upon this OMP vaccine strategy and using *B. cenocepacia* OMPs with an alternative mucosal nanoemulsion adjuvant, resulted in a dose-dependent, nearly sterilizing immunity against *B. cenocepacia* and *B. multivorans* [70].

It is becoming increasingly clear that subunit vaccines that only produce an antibody response neither fully protect nor fully eliminate the pathogen. Therefore, these findings have led to the search for alternative vaccine strategies.

### 5.2. Complex Carbohydrates or Polysaccharides as Vaccine Components

Within the past decade, polysaccharides have been confirmed as important virulence factors and protective antigens against *B. pseudomallei* and *B. mallei* [87,88]. Recently, glycoconjugate vaccine strategies have been explored for various bacterial pathogens and were successful with *Haemophilus influenzae* type B, pneumococcus, and meningococcus [89,90]. These vaccines are attractive because they can induce an antibody response to the polysaccharide and protein by processing both components through a T cell dependent pathway [90,91].

Recently, some labs have been working on synthesizing glycan candidates aimed for incorporation into a glycoconjugate platform vaccine. The first example of successful synthesis was the complex carbohydrate β-Kdo-containing exopolysaccharide, which is produced by *B. pseudomallei* and has been isolated from four of the Bcc species [92]. An additional study in this area successfully synthesized the outer core fragment of *B. multivorans* lipooligosaccharide [93]. Both saccharides have yet to be tested, but are intended as synthesizable cepacia complex-specific glycoconjugate platform. The testing of these newly synthesized glycogens with proteins determined by proteomic analysis could potentially be an effective vaccine and it is definitely an avenue that should be pursued.

Additionally, when considering a glycoconjugate strategy, it is extremely important to keep in mind the *O*-acetyl groups, which are homologous to the native carbohydrate found in Bcc. For many pathogens, *O*-acetyl groups are essential for the development of functional immune responses; therefore, the absence of these residues results in an incomplete immune response [80].

### 5.3. Proteomics

**Figure 1:**
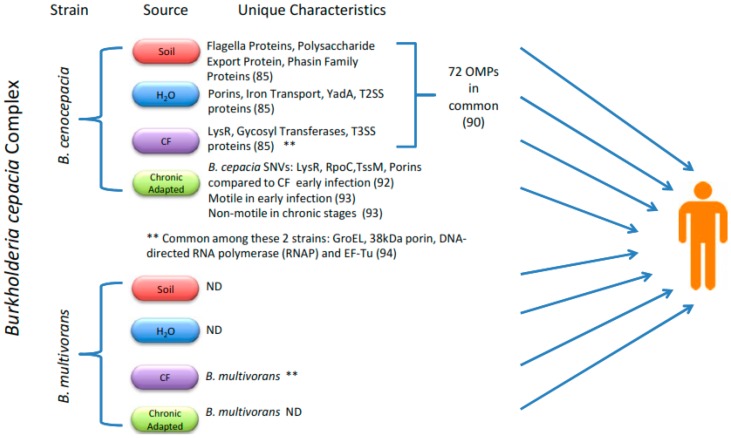
Proteomic analysis showing differences in proteomic expression of Bcc species when cultured in various environments and after adapting to the CF host environment. Here, we highlight some of the differentially expressed proteins resulting from adaptation to the CF or other environments, and the four proteins found to be consistently expressed in *B. cenocepacia* and *B. multivorans* (**).

When advancing the testing of subunit-based vaccines, it is important to consider the differential expression of proteins between environments and through the course of an infection, as well as their overall stability as immunogenic proteins (Figure 1). A recent paper elegantly examined the differential expression of OMPs from *B. cenocepacia* and four different environments: soil, water, plants, and CF sputum. The investigators found 72 proteins expressed among *B. cenocepacia* when it was grown under all four conditions [94]. Growth in water, CF sputum-like environment, and soil resulted in 33, 27, and 20 niche-specific expressed proteins, respectively [94].

When designing a vaccine, we must remember that the aim is to provide an immune response that prevents the establishment of infection. Even though most Bcc infections result from nosocomial outbreaks via person-to-person transmission or by environmental contamination, acquiring an infection from soil or water isolates is still a possibility. Therefore, an overall effective vaccine should harbor not only CF bacterial-specific antigens, but also ubiquitously expressed environmental-related proteins.

It is well accepted that *Burkholderia* species have evolved to establish population heterogeneity that may increase their ability to survive in various environments [95]. Moreover, a single isolate may form heterogenic populations, as seen in a recent report that followed the progression of an endemic *B. cenocepacia* isolate in a single patient over time. The bacteria exhibited several signal nucleotide variations (SNVs), morphological changes, accumulated mutations in *rpoC*, and changes in iron metabolism and transcriptional profiles over time [96]. These data reinforce the importance of adaptation as a successful mechanism among *Burkholderia* species. This study provided information on how an outbreak could stem from a chronically infected individual harboring an adapted *B. cenocepacia*, or a less-adapted bacterium from the environment, and this is a consideration to be kept in mind when developing a vaccine. A more comprehensive study examined the differential gene expression of 25 *B. cenocepacia* patient isolates; contrary to previous observations with *B. cenocepacia*, increased virulence expression and exopolysaccharide synthesis were found, but reduced flagellar gene expression was observed [97]. The latter data helped elucidate the expression profile timeline, and this assisted in the selection of protein candidates that may serve as effective immune targets during early phases of infection and/or infections originating from a chronically adapted Bcc species.

It is important to remember that, while selecting a protein, it would be ideal to find antigens that are represented across multiple Bcc species. In this context, a study compared antigenic proteins from four strains, two *B. cenocepacia* and two *B. multivorans*, and concluded that a concise list of 15 immunogenic proteins are conserved between all four stains. Some of these proteins included GroEL, DNA-directed RNA polymerase, A 38 kDa porin, and EF-Tu [74]. A caveat is that the study methods involved membrane preparation. While only five OMPs were predicted to be membrane-associated, the remaining ten might actually be interacting with the membrane and potentially exposed to the immune system. This scenario could apply to EF-Tu, which is a cytosolic protein, but has been shown to be immunogenic and protective against *B. pseudomallei* [98,99].

## 6. Vaccine Delivery Complications

Because CF patients have excess mucus build-up in the lungs, successful vaccination and treatment have posed a significant problem. Several issues come into play with intranasal vaccination of CF patients; thus, it would be beneficial to investigate alternative routes of delivery. Recently, progress has been reported in the treatment of Bcc infections by using extended-released polymer platforms, which have cellulose as a hydrophobic carrier or natural and synthetic biodegradable polymer [100]. This technology could be utilized to find a platform more compatible with the higher salt concentration found in CF patients' lungs, which could help with the delivery of vaccine materials through the mucus layer [101]. Another promising approach to bypass these difficulties may be oil-water nanoemulsions, a mucosa-specific adjuvant that has demonstrated good delivery and protection [70]. These delivery platforms might be promising approaches when dealing with CF patients, but the key issue is to find a vaccine that elicits the right immune response to reduce bacterial burden and lead to complete pathogen clearance.

## 7. Conclusions and Future Directions

The biggest challenge to overcome with *Burkholderia* infections is identifying a vaccine strategy that produces reduced colonization or sterilizing immunity, meaning the immune system is able to eradicate the pathogen. Sterilizing immunity is important because, once a host is colonized, the Bcc bacteria is able to use several virulence strategies to overcome the immune system, making it difficult to eliminate from the infected lungs and causing compounding problems when the immune system becomes further compromised [32,102,103,104]. It is evident that there are many avenues that can be further investigated, including glycoconjugate, using heat-killed or live attenuated bacteria, as well as extensive Th1 adjuvant testing that can improve subunit-based and OMPs vaccines.

More than a decade ago, a research group investigated adjuvants ornithine lipids and partially degraded LPS from *B. cenocepacia*. They found that the degraded LPS had lower toxicity than traditional LPS, but was able to elicit a more robust immune response compared to that with alum adjuvant. This finding was promising, and this polysaccharide could be tested in combination with OMPs [105]. Alternatively, Bcc-specific glycoconjugate platforms, and possibly extended-release nanoparticles, should be tested to evaluate their ability to elicit a more prominent response in the Th2 biased CF host.

Given the modest efforts toward developing a Bcc vaccine to date, other options should be investigated. An unexplored avenue is the development of live attenuated vaccines. Although there is no clear reason for the lack of advancement in this area, clinical reservations still exist regarding the use of these types of vaccine on immunocompromised patients and the possible complications that could be associated. However, the importance of producing a Bcc vaccine that possesses multiple antigens (perhaps a combination of Th1 and Th2 responses) should be addressed, as well as the development of novel strategies in the construction of live attenuated vaccines with reduced risk to the patient (*i.e.*, multiple gene deletions or short-term surviving strains while maximizing the immune responses elicited).

It is also important to consider that, by limiting our vaccine platform options, we are stunting the growth in this field. We should also consider the development of a therapeutic vaccine candidate that could reduce disease incidence and bacterial persistence with/without combination of antibiotic treatment. However, at the current speed, it may take decades until researchers can unravel effective vaccine strategies that can induce the optimal immune responses required to achieve sterilized immunity.

## Abbreviations

The following abbreviations are used in this manuscript:

Bcc: *Burkholderia cepacia* Complex
CF: Cystic Fibrosis
EPS: Exopolysaccharides
SNVs: Single nucleotide variants
OMP: Outer membrane proteins
